# Metabolomics Study Suggests the Mechanism of Different Types of Tieguanyin (Oolong) Tea in Alleviating Alzheimer’s Disease in APP/PS1 Transgenic Mice

**DOI:** 10.3390/metabo12050466

**Published:** 2022-05-22

**Authors:** Youying Tu, Hyunuk Kang, Eunhye Kim, Jiangfan Yang, Puming He, Yuanyuan Wu, Bo Li, Xiaobo Liu, Junsheng Liu

**Affiliations:** 1 Department of Tea Science, Zhejiang University, Hangzhou 310058, China; youytu@zju.edu.cn (Y.T.); 11616111@zju.edu.cn (H.K.); ehkim@zju.edu.cn (E.K.); pmhe@zju.edu.cn (P.H.); yywu@zju.edu.cn (Y.W.); drlib@zju.edu.cn (B.L.); 2 College of Horticulture, Fujian Agriculture and Forestry University, Fuzhou 350002, China; yjf3001@163.com

**Keywords:** Alzheimer’s disease, metabolomics, gut microbiota, oolong, tea, Tieguanyin

## Abstract

Previously, we found that three types of Tieguanyin tea (Tgy-Q, Tgy-N and Tgy-C) extracts could alleviate Alzheimer’s disease (AD) in a mouse model among which Tgy-C was more effective. In this study, APP/PS1 transgenic mice were used to investigate the metabolomic changes in the feces of mice treated with Tieguanyin tea extracts. Results showed that the profile of fecal metabolites was obviously changed in AD mice. Metabolomics analysis found the effects of Tgy-C, especially its decreasing effect on the fecal metabolites in AD mice—132 of the 155 differential metabolites were decreased. KEGG enrichment revealed that differential metabolites could participate in functional pathways including protein digestion and absorption, biosynthesis of amino acids and ABC transporters. Further comparisons of the metabolites between groups showed that although Tgy-N and Tgy-Q exerted a decreasing effect on the fecal metabolites, Tgy-C was more effective. Moreover, correlation analysis found that the levels of the fecal metabolites were highly correlated with the contents of functional components in tea extracts. Finally, 16S rDNA sequencing presented that Tieguanyin extracts modified the gut microbiota by targeting diverse bacteria. In this study, we investigated the differences of three types of Tieguanyin tea extracts on the fecal metabolites as well as the bacterial community of the gut microbiota in AD mice. The identified differential metabolites and the changed intestinal bacteria might provide potential diagnostic biomarkers for the occurrence and progression of AD.

## 1. Introduction

Alzheimer’s disease (AD) is one of the most common neurodegenerative diseases characterized by cognitive loss, which is mainly manifested as progressive memory dysfunction, cognitive dysfunction, personality abnormalities and language impairment [1]. AD is a disease that typically increases in incidence with age, with an incidence of less than 1% in the 60–64 age group, and the incidence increases exponentially with age. It is estimated that more than 50 million people worldwide are living with dementia, and the number of people affected will exceed 150 million by 2050 [2]. AD has become the fourth leading cause of death after cardiovascular disease, stroke and cancer [3]. With the aging of the population, the improvement of social medical conditions and the extension of the average life expectancy of human beings, AD patients are increasing, bringing severe challenges to the society. Due to the complex pathogenic factors of AD, no theory can give a full explanation to the pathogenesis of AD. Clinical studies have shown that 1% to 5% of cases are caused by gene mutations, and the contributing factors for the rest are unclear [4]. Studies suggest that oxidative stress, inflammation and amyloid-β (Aβ) can contribute to the pathogenicity of AD [5,6,7,8]. Accordingly, many drugs have been developed in clinical trials to improve AD with multiple mechanisms. For example, Tacrolimus can actor as a calcineurin inhibitor to improve synaptic plasticity. The Nicotinic acetylcholine receptors agonist nicotine can improve cholinergic neurotransmission, while Brexpiprazole can enhance dopaminergic neurotransmission. BEY2153 is an Aβ inhibitor that can improve AD conditions by reducing Aβ aggregation toxicity [9,10]. Tea is one of the most popular beverages in the countries of the East and the West, and can be divided into six categories (green tea, white tea, yellow tea, oolong tea, black tea and dark tea) based on the fermentation degree [11]. Polyphenols, flavonoids, caffeine, theanine and other macromolecules are considered to be the major functional compounds of tea, which can exert anti-oxidative, anti-inflammatory, anti-tumor, anti-radiative and other effects. Many studies have suggested the great prospect of tea in managing AD. In 2006, a survey among 1003 old (age > 70) residents in Japan presented the association of a higher consumption of green tea with a lower prevalence of cognitive impairment [12]. The consumption of green tea catechins and black tea theaflavins was reported to be positively associated with language and verbal memory [13]. Another cross-sectional study involving 716 adults aged 55 or older also found that the consumption of black and oolong tea was associated with better cognitive performance [14]. A 3-year cohort study among 5693 residents aged 65 or older in China showed an inverse association between tea drinking and cognitive decline [15]. In a rat model of AD, green tea was found to be more effective in neuroprotection than red and black teas [16].

The similarity of the genome between humans and mice encourages the development and application of mouse models for human diseases, including metabolic disorders. It has long been suspected that the manifestation of heritable disease can differ between individuals due to the heterogeneity of genetic background. Mouse systems with a range of genetic mutations are now permitting a more thorough investigation of human diseases [17]. Our previous research found that three different types of Tieguanyin (Chenxiang, Tgy-C; Qingxiang, Tgy-Q; Nongxiang, Tgy-N), especially Tgy-C, could alleviate AD in APP/PS1 transgenic mouse [18]. In this study, we investigated the effects of three types of Tieguanyin on the fecal metabolites in the mouse model of AD to reveal the mechanisms for the improvement of AD.

## 2. Results

### 2.1. Tieguanyin Treatment Improved the Brain Pathological Conditions of AD Model Mouse

**Figure 1:**
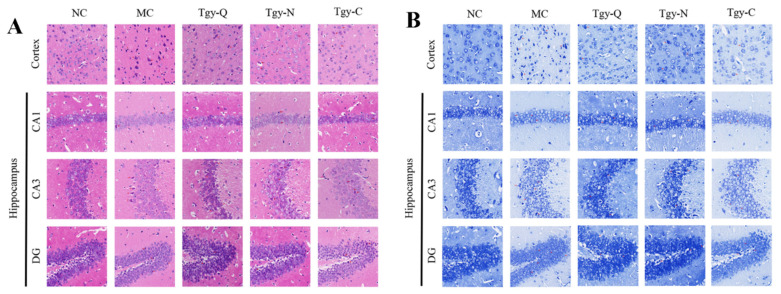
Improvement in cortex and hippocampus of APP/PS1 mice by Tieguanyin extracts. H&E (**A**) and Nissl (**B**) staining of cortex and hippocampus (CA1, CA3 and DG) of wild-type (NC), APP/PS1 (MC) and Tieguanyin-treated APP/PS1 (Tgy-Q, Tgy-N, Tgy-C) mice (×400 magnification).

To study the effects of Tieguanyin extracts on the morphology of the brain, slices of H&E staining were observed with optical microscope. Results (Figure 1A) showed that the cortex and hippocampus (CA1, CA3 and DG) of control mice (NC) were stained homogeneously with normal cell structure; the cell membrane, nucleolus and nuclear membrane were clear. In AD model mice (MC in Figure 1A), the cortex and hippocampus displayed changes in cell morphology. Many cells showed pyknotic nuclei and hyperchromatic cytoplasm while the cell membrane and nuclear membrane were not as clear as NC group. Tieguanyin-treated groups (Tgy-Q, Tgy-N and Tgy-C in Figure 1A) showed much ordered arrangement and clearer membrane borders; the incidence of pyknotic nuclei and hyperchromatic cytoplasm was much lower.

Nissl body is one of the characteristic structures of neurons. To make a further investment on the neurons, the slices were stained with Nissl and then observed with optical microscope. Results showed that when compared with the control group (NC in Figure 1B), cells in the cortex and hippocampus of AD model mice (MC in Figure 1B) were much scattered, and neurons were disordered with reduced number of Nissl bodies. Mice treated with Tieguanyin extracts (Tgy-Q, Tgy-N and Tgy-C in Figure 1B) displayed an improved arrangement of cells and an increased number of Nissl bodies. These results of histological observation clearly demonstrated improved brain pathological conditions of AD model mice by Tieguanyin treatment.

### 2.2. Quality Control for UHPLC-Q-TOF/MS Performance

**Figure 2:**
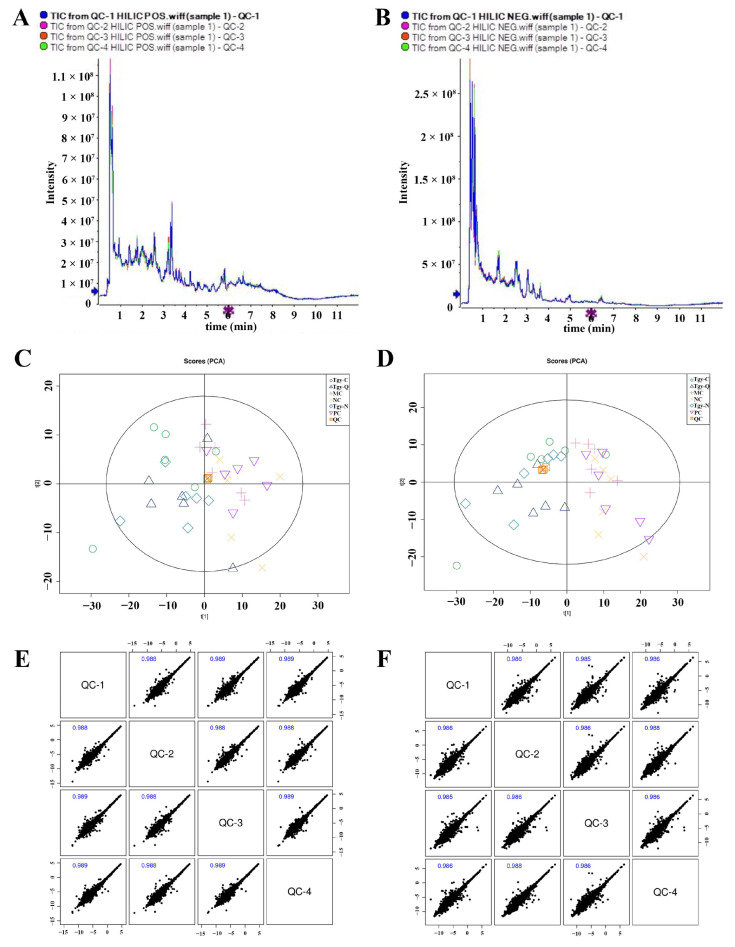
Information of quality control (QC) of UHPLC-Q-TOF/MS performance. Total ion current (TIC) of the QC samples in positive (**A**) and negative (**B**) ion modes; principal component analysis (PCA) of the metabolites in positive (**C**) and negative (**D**) ion modes. Correlation analysis of the QC samples in positive (**E**) and negative (**F**) ion modes.

In this study, mice feces were collected at sacrifice for metabolome test by UHPLC-Q-TOF/MS. All samples were mixed with equal volume to prepare quality control samples (QC). The total ion current (TIC) chromatograms in positive (Figure 2A) and negative (Figure 2B) modes showed that all the peaks of QC were well overlapped, and the intensity and retention time are consistent.

By analyzing all the tested samples with XCMS software, 14,540 positive ions and 12,701 negative ions were detected. Principal component analysis (PCA) of the detected metabolites in positive (Figure 2C) and negative (Figure 2D) ion modes showed that all the QC samples aggregated closely. Moreover, correlation with the Pearson method presented high correlations between the QC samples (Figure 2E,F, correlation coefficient > 0.9). All these results presented good performance of the equipment and high reliability of the results.

### 2.3. The Fecal Metabolome Was Obviously Changed in AD Mice

To explore the influence of AD to the metabolites in mice feces, orthogonal partial least squares discriminant analysis (OPLS-DA) was performed based on the detected ions in model control (MC, APP/PS1 AD mice) and negative control (NC, conventional mice) groups.

**Figure 3:**
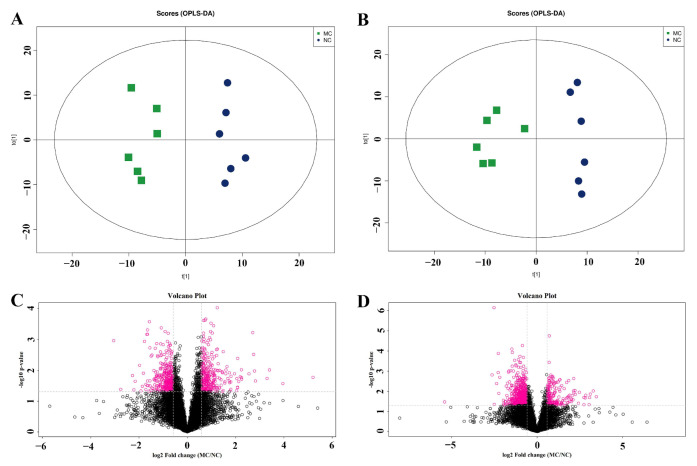
Distinct metabolomic profile in Alzheimer’s disease (AD) mice. Score scatter plot of orthogonal partial least squares discriminant analysis (OPLS-DA) for model control (MC) and negative control (NC) groups in positive (**A**: R2X = 0.397, R2Y = 0.996, Q2 = 0.623) and negative (**B**: R2X = 0.415, R2Y = 0.994, Q2 = 0.571) ion modes. Analysis of differential metabolites between MC and NC mice in positive (**C**) and negative (**D**) ion modes. MC: model control, AD mice; NC: negative control, conventional mice.

Results showed that MC group had distinct metabolomic profiles with NC group both in positive (Figure 3A) and negative (Figure 3B) ion modes. The metabolites were then compared in the volcano plot in positive (Figure 3C) and negative (Figure 3D) ion modes, and the metabolites with fold change (FC) > 1.5 and *p* < 0.05 were marked with pink rings which clearly presented the differences in the metabolites. Then, differential metabolites were identified when VIP (variable importance for the project) >1 and *p* < 0.1 in OPLS-DA test (significant differential metabolites were defined as VIP > 1 and *p* < 0.05). Finally, 33 and 20 differential metabolites were found, respectively, in positive and negative modes, respectively, between MC and NC groups.

**Figure 4:**
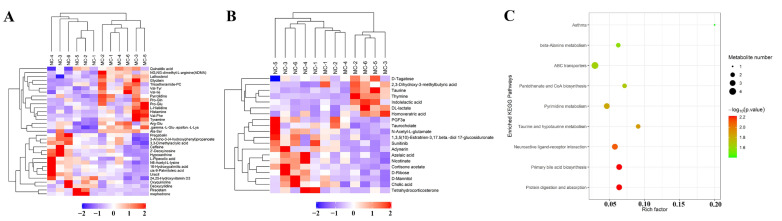
Clustering of the differential metabolites between MC and NC groups and the results of KEGG enrichment. The differential metabolites between MC and NC groups were clustered in positive (**A**) and negative (**B**) ion modes, and all the involved metabolites were enriched based on KEGG database (**C**).

Hierarchical clustering based on these differential metabolites (Figure 4A,B) clearly presented their changes in AD model mice, as well as the difference and relationship between samples. Enrichment with KEGG database (Figure 4C) indicated that these differential metabolites were associated with several pathways, including protein digestion and absorption, primary bile acid biosynthesis, beta-alanine metabolism, etc.

### 2.4. Tgy-C Changed the Profile of Fecal Metabolites in AD Mice

We previously found that Tgy-C could alleviate AD with great efficiency [18]. Then, we compared the fecal metabolites between AD and Tgy-C-treated AD mice to explore the metabolomic mechanisms.

**Figure 5:**
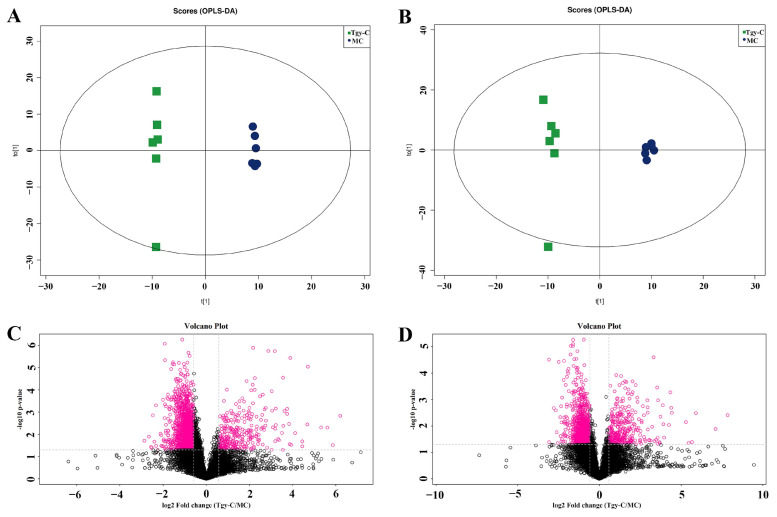
Tgy-C treatment changed the profile of metabolome in AD model mice. Score scatter plot of OPLS-DA for Tgy-C and MC groups in positive ((**A**): R2X = 0.454, R2Y = 0.999, Q2 = 0.748) and negative ((**B**): R2X = 0.497, R2Y = 0.994, Q2 = 0.673) ion modes. Analysis of differential metabolites between Tgy-C and NC mice in positive (**C**) and negative (**D**) ion modes. Tgy-C: Tgy-C-treated AD mice; MC: model control, AD mice.

OPLS-DA showed that both positive (Figure 5A) and negative (Figure 5B) ions were significantly changed in Tgy-C-treated mouse feces. Figure 4D and Figure 5C showed the changes in positive and negative ions between group Tgy-C and group MC, respectively. In Figure 5C,D, there seemed to be more decreased metabolites than increased metabolites. OPLS-DA identified 101 positive and 54 negative ions as differential metabolites (VIP > 1, *p* < 0.1). Among these 155 differential metabolites, 132 were decreased and 23 were increased.

**Figure 6:**
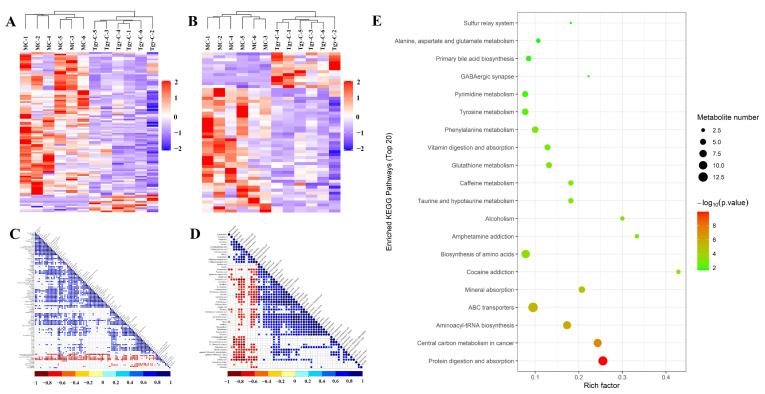
Clustering of the differential metabolites between Tgy-C and MC groups and the results of KEGG enrichment. The differential metabolites between Tgy-C and MC groups were clustered in positive (**A**) and negative (**B**) ion modes. The differential metabolites in positive (**C**) and negative (**D**) ion modes were used for correlation analysis, and all the involved metabolites were enriched based on KEGG database (**E**).

Hierarchical clustering of the differential metabolites of positive (Figure 6A) and negative (Figure 6B) ions further confirmed that Tgy-C tended to decrease the levels of many fecal metabolites. Correlation analysis in positive (Figure 6C) and negative (Figure 6D) ion modes demonstrated that most differential metabolites were highly positively correlated. KEGG enrichment (Figure 6E) revealed that these differential metabolites were involved in protein digestion and absorption, biosynthesis of amino acids, ABC transporters, central carbon metabolism in cancer, tyrosine metabolism, etc.

**Figure 7:**
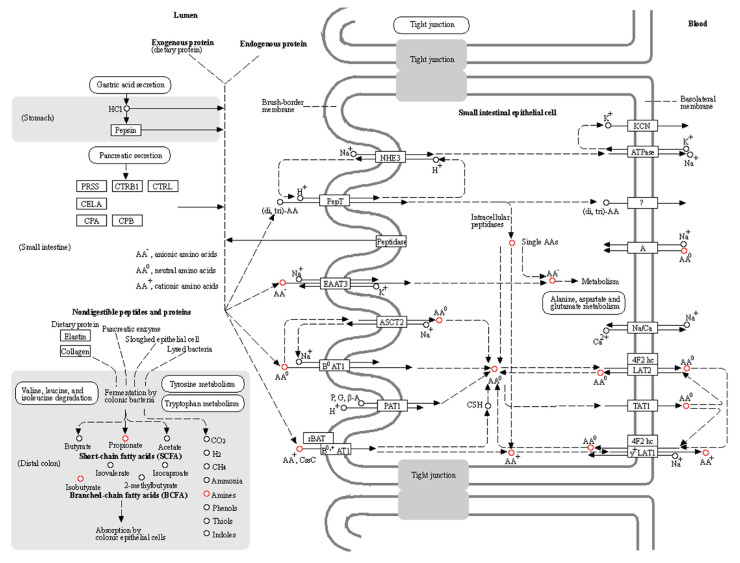
Differential metabolites between Tgy-C and MC groups in protein digestion and absorption pathway.

The KEGG pathway map of protein digestion and absorption (Figure 7) showed the metabolites involved in this pathway. By detailing the information (from OPLS-DA) of the changes in the differential metabolites in protein digestion and absorption pathway (Table 1), we presented that only L-Tyrosine showed an increase in the Tgy-C group, while the other 11 metabolites were all decreased.

The information of the differential metabolites in biosynthesis of amino acids (Table S2) and ABC transporters (Table S3) also showed that more metabolites were decreased. These results revealed that Tgy-C significantly changed the fecal metabolites in AD mice, and especially decreased the metabolites associated with various functional pathways. Notably, many amino acids in the pathways of protein digestion and absorption and biosynthesis of amino acids were decreased in Tgy-C group. It was reported that insufficient protein intake was related to the decrements of the risks of Alzheimer’s disease and related dementias [19]. It deserves further studies to determine whether Tgy-C improves AD by reducing the availability or promoting the digestion and absorption of protein and amino acids.

### 2.5. Different Types of Tieguanyin Exerted Distinct Effects on the Fecal Metabolites Which Correlated with the Contents of Functional Components in the Tea Extracts

Because Tgy-C was more effective than other types of Tieguanyin in alleviating AD, we further compared their difference in modulating the fecal metabolites. OPLS-DA (Figure S1) found that Tgy-C turned AD model mice into different metabolomic profiles with other types of Tieguanyin (Tgy-N and Tgy-Q). As Tgy-C could modulate metabolites associated with protein digestion and absorption (Table 1), biosynthesis of amino acids (Table S2) and ABC transporters (Table S3), further analysis found that Tgy-N (Table 2, Tables S4 and S5) and Tyg-Q (Table 3, Tables S6 and S7) also changed the metabolites associated with these pathways, but in different ways from Tyg-C.

As mentioned above, 132 of the 155 differential metabolites between Tgy-C and MC groups were decreased. Then, we collected the information of the differential metabolites between groups. Results showed that there were 53 differential metabolites between MC and NC groups (Table 4).

**Figure 8:**
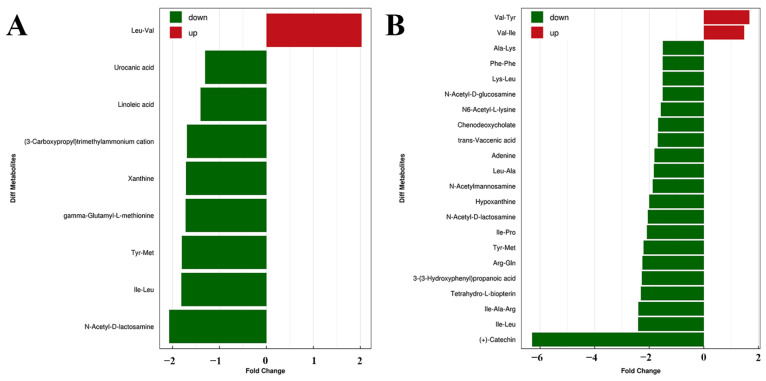
Differential metabolites between Tgy-C and other tea-treated groups (**A**: Tgy-C vs. Tgy-N; **B**: Tgy-C vs. Tgy-Q) in the positive ion mode.

Among them, 24 were increased and 29 were decreased in MC. When compared with MC group, Tgy-C, Tgy-Q and Tgy-N generated 155, 123 and 171 differential metabolites, respectively. Among the 155 differential metabolites between Tgy-C and MC groups, 132 were decreased. When compared with the MC group, Tgy-Q and Tgy-N groups also found more decreased differential metabolites than those of increased (Table 4). These results showed the strong capability of Tieguanyin in down-regulating the fecal metabolites in AD mice. To further probe into the ability of different types of Tieguanyin in modulating the metabolome, comparisons were made between Tgy-Q, Tgy-N and Tgy-C groups. Results showed that among the 46 differential metabolites between Tgy-C and Tgy-Q groups, 39 were decreased; 22 of the 34 differential metabolites between Tgy-C and Tgy-N groups were decreased. Changes in the positive ions were compared and shown in Figure 8. These results showed that Tgy-C was more prominent than other types of Tieguanyin in decreasing the fecal metabolites in AD model mouse.

**Figure 9:**
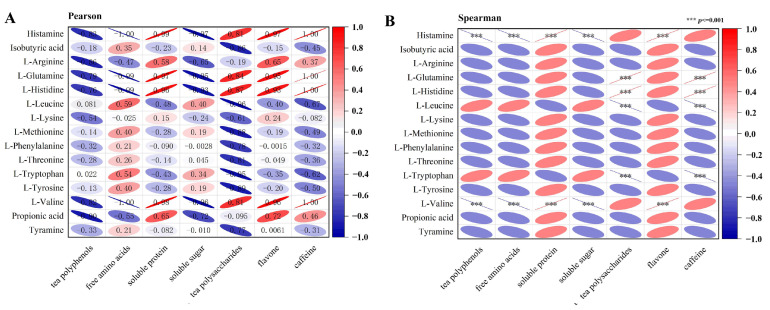
Correlation analysis between the metabolites in feces and the components in tea extracts. Pearson (**A**) and Spearman (**B**) analyses were performed to analyze the correlation between the metabolites in feces and the functional components in tea extracts. In the Spearman test, the metabolites and the components were considered to be significantly correlated when *p* ≤ 0.05 (*** *p* ≤ 0.001).

Correlation analysis with Pearson test (Figure 9A) showed that the content of tea polyphenols was negatively correlated with the levels of the tested metabolites, which presented high correlation coefficient with histamine (−0.83), L-arginine (−0.86), L-glutamine (−0.79), L-valine (−0.82) and Tyramine (−0.90). Soluble sugar, tea polysaccharides, flavone and caffeine were all found to positively correlate with L-glutamine and L-histidine (correlation coefficient > 0.84). Spearman test (Figure 9B) presented that the contents of tea polyphenols were significantly and negatively correlated with histamine and L-valine (*p* ≤ 0.001). Histamine and L-valine were also found to be significantly correlated with free amino acids, soluble protein and soluble sugar (*p* ≤ 0.001, Figure 9B). L-glutamine and L-histidine were positively correlated with tea polysaccharides and caffeine, while L-leucine was negatively correlated with them (*p* ≤ 0.001, Figure 9B).

### 2.6. Different Effects of Tieguanyin on the Gut Microbiota

**Figure 10:**
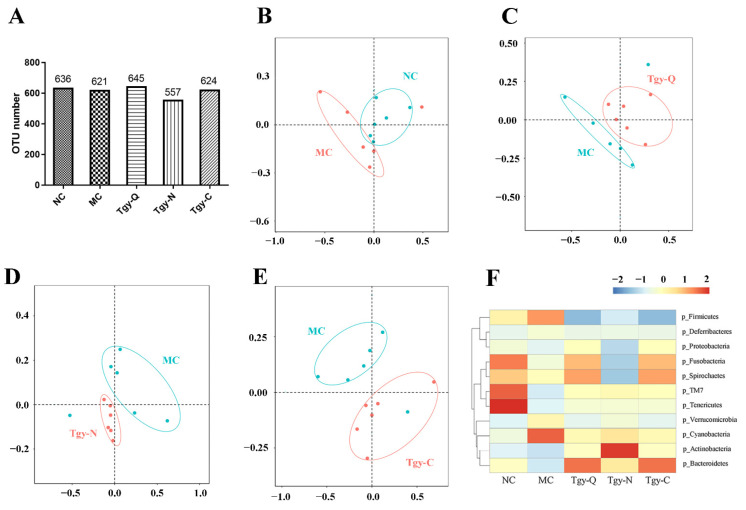
Tieguanyin extracts changed the profile of gut microbiota. Mice feces were collected for sequencing, and the community structure of the gut microbiota was studied. (**A**) The number of OTU in different groups. (**B**–**E**) Non-metric multidimensional scaling (NMDS) analysis of the gut microbiota in OTU level between groups. (**F**) Correlation analysis of the gut microbiota at the phylum level (**F**).

The gut microbiota can affect AD through the gut–microbiome–brain axis [20]. 16S rDNA sequencing showed that both AD (MC in Figure 10A) and Tieguanyin (Tgy-Q, Tgy-N and Tgy-C in Figure 10A) changed the OTU number in mice feces. Further analysis with NMDS showed distinct profiles of the gut microbiota in control (NC) and AD (MC) mice (Figure 10B).

Moreover, all types of Tieguanyin extracts (Tgy-Q, Tgy-N and Tgy-C in Figure 10C, 10D and 10E, respectively) changed the profile of the gut microbiota of AD mice. The heat map at the phylum level (Figure 10F) showed that Tieguanyin could change the abundance of the gut bacteria with different targets. Firmicutes were enriched in the MC group and all Tieguanyin-treated groups had a much lower relative abundance. Tgy-N tended to increase the relative abundance of Actinobacteria and decrease the relative abundance of Proteobacteria, Fusobacteria and Spirochaetes. Tgy-Q and Tgy-C tended to decrease the relative abundance of Firmicutes and increase the relative abundance of Bacteroidetes and Spirochaetes.

## 3. Discussion

Tea (*Camellia sinensis*) has originated in China as a kind of medicine about 2727 B.C [21]. Nowadays, drinking tea is becoming more and more popular across the world because of its health benefits. Flavonoids, caffeine and theanine are recognized as the major bioactive macromolecules, which are linked to the prevention of many diseases, such as cardiovascular diseases, metabolic diseases and immune system disorders, in many studies [21]. Alzheimer’s disease (AD) is one of the most complicated neurodegenerative diseases characterized by progressive dementia and deterioration of cognitive function. Many studies have revealed the great potential of tea functional components in preventing AD. Oxidative stress and inflammation are considered to be important causative mechanisms of AD, and catechins could exert anti-oxidative and anti-inflammatory effects with great efficiency to become a neuroprotective agent [22,23]. In APP/PS1 transgenic mice, epigallocatechin-3-gallate (EGCG) improved cognitive deficits through neuroprotective, anti-amyloidogenic and anti-inflammatory mechanisms [24]. In another AD model of SAMP8 mouse, long-term consumption of EGCG showed an improvement in spatial learning and memory [25]. In in vitro studies, L-theanine was found to be neuroprotective on human neuroblastoma cells [26]. In a mouse model, L-theanine improved memory by the suppression of ERK/p38 and NF-κB [27]. These findings have encouraged people to explore the anti-AD effects and the involved mechanisms of drinking tea.

Tieguanyin is a famous oolong tea comes from Anxi County, Fujian Province, China. It is widely welcomed because of its unique fragrance and health benefits [28]. Our group previously found that different types of Tieguanyin (Tgy-Q, Tgy-N and Tgy-C) could alleviate AD, especially the type of Tgy-C [18]. In this study, we explored the effects of different types of Tieguanyin on the fecal metabolites in APP/PS1 transgenic mouse model of AD.

First, we compared the fecal metabolites between AD and conventional mice. OPLS-DA found 53 differential metabolites between MC (AD mice) and NC (conventional mice) groups (Table 4). This result indicated that the metabolism of the gut microbes was altered in AD mice or the structure of the gut microbes was changed. The identified differential metabolites might be biomarkers for AD mice and might act as indicators for the occurrence or progression of AD. Among the differential metabolites between MC and NC groups, 24 were increased and 29 were decreased (Table 4). Comparison between Tgy-C and MC groups found more differential metabolites, among which 23 were increased and 132 were decreased (Table 4). These results showed the great capability of Tgy-C in modulating the metabolome, and also indicated the tendency of Tgy-C in decreasing the metabolites. Further comparisons between other Tieguanyin-treated groups (Tgy-Q and Tgy-N) and the MC group showed that most of the differential metabolites were decreased (Table 4). It deserves further studies to probe into whether drinking Tieguanyin could alleviate AD by restricting the metabolism of the intestinal bacteria. Tgy-C was previously found to be more effective in alleviating AD, and in this study, Tgy-C was found to be more effective in decreasing the levels of the fecal metabolites (Table 1). So, it is possible that the decrease in fecal metabolites might favor the improvement of AD.

Studies have revealed that insufficient protein could reduce the risks of developing Alzheimer’s disease and related dementias [19]. In this study, we also found a decrease in many amino acids in the feces of mice treated with Tieguanyin tea extracts (Table 1, Table 2 and Table 3, Tables S2, S4 and S6). Additionally, the efficiency of Tgy-C in decreasing the amino acids (Table 2 and Table 3) more than other Tieguanyin tea extracts might at least partly contribute to its prominent capability to alleviate AD. D-amino acids were considered to play important roles in AD [29]. In this study, we found that many L-amino acids were modulated by Tieguanyin tea extracts (Table 1, Table 2 and Table 3), indicating critical roles of L-amino acids in AD.

KEGG enrichment with the differential metabolites between MC and NC groups presented functional alteration of the fecal metabolome in AD mice (Figure 4C). This provided further evidence for the existence of the gut–brain axis from the aspect of microbial metabolomics. Enrichment based on the differential metabolites between Tgy-C and MC groups showed that Tgy-C treatment affected functional pathways including protein digestion and absorption, caffeine metabolism and biosynthesis of amino acids (Figure 6E).

The changes in the differential metabolites involved in these pathways (Table 1, Table 2, Table 3 and Tables S1–S6) further confirmed the decreasing effects of Tieguanyin on the fecal metabolites. Correlation analysis showed that the differential metabolites between Tgy-C and MC groups were highly positively correlated (Figure 6C,D). We believe that the mechanism underlying the high correlation of these metabolites is the modified bacterial community. Then, mice feces were collected for sequencing. Tgy-Q, Tgy-N and Tgy-C changed the gut microbiota into distinct profiles with the MC group (Figure 9C–E). Analysis at phylum level showed that different types of Tieguanyin had different targets of the gut bacteria. Further studies are still needed to investigate the community changes at the species level, as well as the functional changes, of the gut microbiota by using metagenomic sequencing, which can be related back to the changes in the metabolites and the improvement of AD.

In this study, we compared the effects of different types of Tieguanyin tea extracts on the fecal metabolites in a mouse model of AD using UHPLC-Q-TOF/MS. Results showed that the metabolome of AD model mouse was obviously changed, and Tieguanyin tea extracts (Tgy-C, Tgy-N and Tgy-Q) exerted different effects on the fecal metabolome in the AD model mice. OPLS-DA found the differential metabolites between Tieguanyin-treated and AD model mice, as well as within Tieguanyin-treated groups. By comparing the differential metabolites between groups, Tieguanyin tea extracts were found to exert decreasing effects on the fecal metabolites, especially the Tgy-C. The changes in the fecal metabolites might contribute to the improvement of AD, and further studies are still needed to reveal the changes in the gut microbiota in Tieguanyin tea extract-treated AD mice underlying the changes in the fecal metabolites and the improvement of AD.

## 4. Materials and Methods

### 4.1. Materials and Chemicals

Different types of Tieguanyin tea (Tgy-Q, Tgy-N and Tgy-C) were kindly provided by Mr. Yuede Wei of Fujian Anxi Qishan Weiyin Famous Tea Co., Ltd. Standards of caffeine, gallic acid (GA) and tea catechins used for high-performance liquid chromatography (HPLC; from SHIMADZU, Kyoto, Japan; data were processed with LC solution V1.26) were purchased from Sigma-Aldrich (St. Louis, MO, USA).

### 4.2. Preparation Characterization of Tieguanyin Extracts

Tieguanyin samples were powdered and extracted 30 times (*v*/*v*) with boiling distilled water for 30 min, and stirred with a glass rod every 10 min. After vacuum filtration, samples were concentrated with rotary evaporator. Samples were stored at −20 °C until use.

Tea polyphenols, flavones, free amino acids, soluble sugar and tea polysaccharides were detected as previously reported [30,31,32,33]. The contents of caffeine, GA and tea catechins were analyzed with high-performance liquid chromatography (HPLC) [34]. The content of soluble protein was determined by Coomassie Brilliant Blue method [35]. The composition of different samples was shown in Table S1.

### 4.3. Animal Experiment

APP/PS1 mouse is a well-established and widely used double transgenic model of AD [36]. Thirty male APP/PS1 (APPswe, PSEN1dE9) adult mice (6 months) with a C57BL/6J background and six male C57BL/6J adult mice were purchased from Shanghai Model Organisms Centre (Shanghai, China). All mice were housed under controlled standard barrier conditions (temperature 23 ± 2 °C, humidity 55% ± 5, and 12 h light–dark cycle) at the Animal Centre of Zhejiang Chinese Medical University. After acclimatization for 1 week, mice were grouped and treated as follows: negative control (NC), C57BL/6J mice (*n* = 6), gavaged with sterilized water; model control (MC), APP/PS1 mice (*n* = 6), gavaged with sterilized water; Tgy-C, APP/PS1 mice (*n* = 6), gavaged with Tgy-C (1000 mg/kg/d); Tgy-N, APP/PS1 mice (*n* = 6), gavaged with Tgy-N (1000 mg/kg/d); Tgy-Q, APP/PS1 mice (*n* = 6), gavaged with Tgy-Q (1000 mg/kg/d); positive control (PC), APP/PS1 mice (*n* = 6), gavaged with Donepezil (1 mg/kg/d). After treatment for 12 weeks, mice feces were collected and stored at −80 °C until use.

### 4.4. Paraffin Section, H&E and Nissl Staining

After sacrifice, the mice brains were removed and fixed with 4% paraformaldehyde, which was followed by dehydration, clearing and paraffin wax immersion. Then, the brain tissue was cut into 5 μm-thick sections and subsequently stained with H&E and Nissl.

### 4.5. Metabolomics Analysis

To extract metabolites from mice feces, 1 mL cold extraction solvent methanol/acetonitrile/H_2_O (2:2:1, *v*/*v*/*v*) was added to 80 mg sample, and adequately vortexed. After vortexing, the samples were incubated on ice for 20 min, and then centrifuged at 14,000× *g* for 20 min at 4 °C. The supernatant was collected and flowed through a 96-well protein precipitation plate, and then the elution was collected and dried in a vacuum centrifuge at 4 °C. For UHPLC-Q-TOF/MS analysis, the samples were re-dissolved in 100 μL acetonitrile/water (1:1, *v*/*v*) solvent.

For untargeted metabolomics of polar metabolites, extracts were analyzed using a quadrupole time-of-flight mass spectrometer (Sciex TripleTOF 6600) coupled to hydrophilic interaction chromatography via electrospray ionization in Shanghai Applied Protein Technology Co., Ltd. LC separation was on an ACQUIY UPLC BEH amide column (2.1 mm × 100 mm, 1.7µm particle size (waters, Ireland) using a gradient of solvent A (25 mM ammonium acetate and 25 mM ammonium hydroxide in water) and solvent B (acetonitrile). The gradient was 85% B for 1 min and was linearly reduced to 65% in 11 min, and then was reduced to 40% in 0.1 min and kept for 4 min, and then increased to 85% in 0.1 min, with a 5 min re-equilibration period employed. Flow rate was 0.4 mL/min, column temperature was 25 °C, auto sampler temperature was 5 °C and injection volume was 2 µL. The mass spectrometer was operated in both negative and positive ionization modes. The ESI source conditions were set as follows: Ion Source Gas1 (Gas1) as 60, Ion Source Gas2 (Gas2) as 60, curtain gas (CUR) as 30, source temperature: 600 °C and IonSpray Voltage Floating (ISVF) ± 5500 V. In MS acquisition, the instrument was set to acquire over the *m*/*z* range 60–1000 Da, and the accumulation time for TOF MS scan was set at 0.20 s/spectra. In auto MS/MS acquisition, the instrument was set to acquire over the m/z range 25–1000 Da, and the accumulation time for product ion scan was set at 0.05 s/spectra. The product ion scan is acquired using information-dependent acquisition (IDA) with high sensitivity mode selected. The parameters were set as follows: the collision energy (CE) was fixed at 35 V with ±15 eV; declustering potential (DP), 60 V (+) and −60 V (−); exclude isotopes within 4 Da; and candidate ions to monitor per cycle: 10.

The raw MS data (wiff.scan files) were converted to MzXML files using ProteoWizard MSConvert before importing into freely available XCMS software. For peak picking, the following parameters were used: cent Wave *m*/*z* = 25 ppm, peak width = c (10, 60) and prefilter = c (10, 100). For peak grouping, bw = 5, mzwid = 0.025 and minfrac = 0.5 were used. In the extracted ion features, only the variables having more than 50% of the nonzero measurement values in at least one group were kept. Compound identification of metabolites by LC-MS/MS spectra with an in-house database established with available authentic standards. After normalized to total peak intensity, the processed data were uploaded into before importing into SIMCA-P (version 14.1, Umetrics, Umea, Sweden), where it was subjected to multivariate data analysis, including Pareto-scaled principal component analysis (PCA) and orthogonal partial least squares discriminant analysis (OPLS-DA). The 7-fold cross-validation and response permutation testing was used to evaluate the robustness of the model. The variable importance in the projection (VIP) value of each variable in the OPLS-DA model was calculated to indicate its contribution to the classification. Significance was determined using an unpaired Student’s *t* test. VIP value >1 and *p* < 0.1 was considered to be different; VIP value >1 and *p* < 0.05 was considered as statistically significant.

Bioinformatics analysis was performed with the help of the platform APTCloud (http://cloud.aptbiotech.com/#/main-page, accessed on 17 July 2021). For KEGG pathway annotation, the metabolites were blasted against the online Kyoto Encyclopedia of Genes and Genomes (KEGG) database to retrieve their COs and were subsequently mapped to pathways in KEGG11. The corresponding KEGG pathways were extracted. To further explore the impact of differentially expressed metabolites, enrichment analysis was performed. KEGG pathway enrichment analyses were applied based on the Fisher’ exact test, considering the whole metabolites of each pathway as background dataset. Additionally, only pathways with *p*-values under a threshold of 0.05 were considered as significantly changed pathways. For hierarchical clustering, Cluster 3.0 (http://bonsai.hgc.jp/~mdehoon/software/cluster/software.htm, accessed on 17 July 2021) and the Java Treeview software (http://jtreeview.sourceforge.net, accessed on 17 July 2021) were used. Euclidean distance algorithm for similarity measure and average linkage clustering algorithm (clustering uses the centroids of the observations) for clustering were selected when performing hierarchical clustering. The correlation analysis between the metabolites and components in tea extracts was performed with OriginPro (2022).

### 4.6. 16S rDNA Sequencing and Bioinformatics Analysis

Mice feces were collected before sacrifice. After extraction and purification of the total DNA, the V3-V4 region of 16S rDNA was amplified by PCR. After agarose gel electrophoresis, gel extraction and quantification, the sequencing library was prepared and sequenced on Illumina HiSeq. Bioinformatics analysis was performed on APT Cloud (Shanghai Applied Protein Technology Co. Ltd., http://cloud.aptbiotech.com, accessed on 17 July 2021): the operational taxonomic units (OTUs) were identified and taxonomically classified. Non-metric multidimensional scaling (NMDS) analysis was performed between groups at the OTU level. Then, the relative abundance of bacteria among all the tested groups was compared at the phylum level and presented in the heat map.

## Figures and Tables

**Table 1:** Differential metabolites between Tgy-C and MC groups in the pathway of protein digestion and absorption.

Description	VIP	Fold Change	*p*-Value
L-Threonine	1.1	0.55	5.9 × 10^−6^
L-Leucine	4.4	0.56	5.3 × 10^−4^
L-Methionine	2.8	0.46	1.4 × 10^−3^
L-Phenylalanine	5.5	0.56	1.9 × 10^−3^
Tyramine	9.1	0.56	2.2 × 10^−3^
L-Tryptophan	1.7	0.57	5.0 × 10^−2^
L-Tyrosine	2.3	1.38	9.9 × 10^−2^
Isobutyric acid	6.2	0.33	8.1 × 10^−6^
L-Alanine	2.5	0.48	1.5 × 10^−3^
L-Lysine	2.6	0.28	9.6 × 10^−3^
L-Glutamate	3.1	0.55	2.3 × 10^−2^
Propionic acid	1.3	0.60	4.9 × 10^−2^

**Table 2:** Differential metabolites between Tgy-N and MC groups in the pathway of protein digestion and absorption.

Metabolites	Tgy-N vs. MC			Tgy-N vs. Tgy-C
	VIP	Fold Change	*p*-Value	Fold Change
L-Threonine	1.2	0.50	3.5 × 10^−4^	0.92
L-Phenylalanine	6.7	0.49	2.4 × 10^−3^	0.89
Tyramine	11	0.49	2.6 × 10^−3^	1.01
L-Methionine	2.8	0.45	3.0 × 10^−3^	1.00
L-Leucine	4.3	0.59	3.2 × 10^−3^	0.95
Histamine	1.5	0.41	1.8 × 10^−2^	0.88
L-Tyrosine	3.1	0.55	2.0 × 10^−2^	0.88
L-Glutamate	2.1	0.55	3.1 × 10^−2^	0.95
L-Histidine	1.8	0.52	4.5 × 10^−2^	0.73
L-Arginine	14	0.44	8.6 × 10^−2^	0.64
L-Tryptophan	2.1	0.58	9.4 × 10^−2^	1.01
Isobutyric acid	5.9	0.25	1.6 × 10^−7^	0.75
L-Alanine	2.4	0.45	2.7 × 10^−4^	0.63
Propionic acid	1.6	0.40	2.2 × 10^−3^	0.67
L-Valine	1.3	0.29	2.3 × 10^−3^	0.51
L-Lysine	2.5	0.22	6.1 × 10^−3^	0.80
L-Glutamine	1.6	0.44	9.5 × 10^−2^	0.72

**Table 3:** Differential metabolites between Tgy-Q and MC groups in the pathway of protein digestion and absorption.

Metabolites	Tgy-Q vs. MC			Tgy-Q vs. Tgy-C
	VIP	Fold Change	*p*-Value	Fold Change
Histamine	1.1	0.51	2.5 × 10^−3^	0.73
L-Arginine	5.5	0.39	3.1 × 10^−2^	1.05
L-Histidine	1.8	0.50	4.8 × 10^−2^	0.93
L-Tryptophan	1.7	0.62	9.0 × 10^−2^	1.08
L-Valine	1.4	0.30	1.3 × 10^−3^	0.53
L-Lysine	2.4	0.36	2.1 × 10^−2^	1.12
Propionic acid	1.5	0.60	2.5 × 10^−2^	1.01
L-Threonine	1.4	0.62	5.9 × 10^−2^	1.34
L-Glutamine	1.6	0.44	6.9 × 10^−2^	0.71

**Table 4:** Number of the differential metabolites between groups and their changes.

Comparisons	POS	NEG	Total
MC vs. NC	33 (17↑, 16↓)	20 (7↑, 13↓)	53 (24↑, 29↓)
Tgy-C vs. MC	101 (11↑, 90↓)	54 (12↑, 42↓)	155 (23↑, 132↓)
Tgy-Q vs. MC	81 (28↑, 53↓)	42 (17↑, 25↓)	123 (45↑, 78↓)
Tgy-N vs. MC	111 (26↑, 85↓)	60 (12↑, 48↓)	171 (38↑, 133↓)
Tgy-C vs. Tgy-Q	36 (4↑, 32↓)	10 (3↑, 7↓)	46 (7↑, 39↓)
Tgy-C vs. Tgy-N	22 (5↑, 17↓)	12 (7↑, 5↓)	34 (12↑, 22↓)
Tgy-N vs. Tgy-Q	20 (4↑, 16↓)	9 (3↑, 6↓)	29 (7↑, 22↓)

## Data Availability

The data presented in this study are available on request from the corresponding author.

## Supplementary Materials

The following supporting information can be downloaded at: https://www.mdpi.com/article/10.3390/metabo12050466/s1, Figure S1: Tgy-C treatment showed different effects on the metabolome with Tgy-N and Tgy-Q in AD mice; Table S1: The main chemical components of Tieguanyin tea extracts; Table S2: Different metabolites between Tgy-C and MC groups in the pathway of biosynthesis of amino acids; Table S3: Different metabolites between Tgy-C and MC groups in the pathway of ABC transporters; Table S4: Different metabolites between Tgy-N and MC groups in the pathway of biosynthesis of amino acids; Table S5: Different metabolites between Tgy-N and MC groups in the pathway of ABC transporters; Table S6: Different metabolites between Tgy-Q and MC groups in the pathway of biosynthesis of amino acids; Table S7: Different metabolites between Tgy-Q and MC groups in the pathway of ABC transporters.
