# Supplementary material for: Metabolomics Study Suggests the Mechanism of Different Types of Tieguanyin (Oolong) Tea in Alleviating Alzheimer’s Disease in APP/PS1 Transgenic Mice
Source: Metabolites. 2022 May 22;12(5):466. doi: 10.3390/metabo12050466 (PMC9142883; doi:10.3390/metabo12050466)

**Table S1.** The main chemical components of Tieguanyin tea extracts.

| Components | Percentage (%) | | |
|---|---|---|---|
| | Tgy-C | Tgy-Q | Tgy-N |
| tea polyphenols | 36.82 | 43.72 | 39.64 |
| free amino acids | 4.79 | 6.41 | 6.26 |
| soluble protein | 10.47 | 8.86 | 9.21 |
| soluble sugar | 27.81 | 36.20 | 33.65 |
| tea polysaccharides | 5.74 | 4.86 | 3.73 |
| flavone | 3.21 | 2.41 | 2.65 |
| caffeine | 7.49 | 7.13 | 7.12 |

**Table S2.** Different metabolites between Tgy-C and MC groups in the pathway of biosynthesis of amino acids.

| Description | VIP | Fold Change | P-value |
|---|---|---|---|
| L-Threonine | 1.1 | 0.55 | 5.9E-06 |
| L-Leucine | 4.4 | 0.56 | 5.3E-04 |
| L-Methionine | 2.8 | 0.46 | 1.4E-03 |
| L-Phenylalanine | 5.5 | 0.56 | 1.9E-03 |
| L-Tryptophan | 1.7 | 0.57 | 5.0E-02 |
| L-Citrulline | 2.3 | 0.69 | 7.4E-02 |
| L-Tyrosine | 2.3 | 1.38 | 9.9E-02 |
| L-Alanine | 2.5 | 0.48 | 1.5E-03 |
| L-Lysine | 2.6 | 0.28 | 9.6E-03 |
| L-Glutamate | 3.1 | 0.55 | 2.3E-02 |

**Table S3.** Different metabolites between Tgy-C and MC groups in the pathway of ABC transporters.

| Description | VIP | Fold Change | P-value |
|---|---|---|---|
| L-Threonine | 1.1 | 0.55 | 5.9E-06 |
| L-Leucine | 4.4 | 0.56 | 5.3E-04 |
| L-Phenylalanine | 5.5 | 0.56 | 1.9E-03 |
| Riboflavin | 1.3 | 0.48 | 8.3E-03 |
| Thiamine | 13 | 0.57 | 2.0E-02 |
| N-Acetyl-D-glucosamine | 1.5 | 0.74 | 3.5E-02 |
| Deoxycytidine | 1.7 | 1.58 | 4.2E-02 |
| Taurine | 1.0 | 0.30 | 6.2E-02 |
| L-Alanine | 2.5 | 0.48 | 1.5E-03 |
| L-Lysine | 2.6 | 0.28 | 9.6E-03 |
| 2'-Deoxyuridine | 5.9 | 0.38 | 1.6E-02 |
| L-Glutamate | 3.1 | 0.55 | 2.3E-02 |
| Deoxyinosine | 5.7 | 0.41 | 3.5E-02 |

**Table S4.** Different metabolites between Tgy-N and MC groups in the pathway of biosynthesis of amino acids.

| Metabolites | VIP | Tgy-N vs MC Fold Change | P-value | Tgy-N vs Tgy-C Fold Change |
|---|---|---|---|---|
| L-Threonine | 1.2 | 0.50 | 3.5E-04 | 0.92 |
| L-Phenylalanine | 6.7 | 0.49 | 2.4E-03 | 0.89 |
| L-Methionine | 2.8 | 0.45 | 3.0E-03 | 1.00 |
| L-Leucine | 4.3 | 0.59 | 3.2E-03 | 0.95 |
| L-Tyrosine | 3.1 | 0.55 | 2.0E-02 | 0.88 |
| L-Glutamate | 2.1 | 0.55 | 3.1E-02 | 0.95 |
| L-Citrulline | 2.4 | 0.65 | 3.1E-02 | 0.94 |
| L-Histidine | 1.8 | 0.52 | 4.5E-02 | 0.73 |
| L-Arginine | 14 | 0.44 | 8.6E-02 | 0.64 |
| L-Tryptophan | 2.1 | 0.58 | 9.4E-02 | 1.01 |
| L-Alanine | 2.4 | 0.45 | 2.7E-04 | 0.63 |
| L-Valine | 1.3 | 0.29 | 2.3E-03 | 0.51 |
| L-Lysine | 2.5 | 0.22 | 6.1E-03 | 0.80 |
| L-Glutamine | 1.6 | 0.44 | 9.5E-02 | 0.72 |

**Table S5.** Different metabolites between Tgy-N and MC groups in the pathway of ABC transporters.

| Metabolites | Tgy-N vs MC | | | Tgy-N vs Tgy-C |
|---|---|---|---|---|
| | VIP | Fold Change | P-value | Fold Change |
| L-Threonine | 1.2 | 0.50 | 3.5E-04 | 0.92 |
| Thiamine | 16.5 | 0.50 | 1.6E-03 | 0.87 |
| L-Phenylalanine | 6.7 | 0.49 | 2.4E-03 | 0.89 |
| L-Leucine | 4.3 | 0.59 | 3.2E-03 | 0.95 |
| Riboflavin | 1.2 | 0.50 | 1.2E-02 | 1.04 |
| L-Glutamate | 2.1 | 0.55 | 3.1E-02 | 0.95 |
| Deoxycytidine | 1.7 | 1.60 | 3.7E-02 | 0.99 |
| L-Histidine | 1.8 | 0.52 | 4.5E-02 | 0.73 |
| N-Acetyl-D-glucosamine | 1.4 | 2.09 | 8.5E-02 | 1.13 |
| L-Arginine | 14 | 0.44 | 8.6E-02 | 0.64 |
| L-Alanine | 2.4 | 0.45 | 2.7E-04 | 0.63 |
| L-Valine | 1.3 | 0.29 | 2.3E-03 | 0.51 |
| 2'-Deoxyuridine | 6.4 | 0.24 | 2.7E-03 | 0.63 |
| L-Lysine | 2.5 | 0.22 | 6.1E-03 | 0.80 |
| Deoxyinosine | 5.6 | 0.39 | 2.8E-02 | 0.95 |
| D-Mannose | 6.4 | 0.56 | 5.1E-02 | 0.71 |
| Taurine | 7.4 | 0.36 | 6.8E-02 | 1.37 |
| L-Glutamine | 1.6 | 0.44 | 9.5E-02 | 0.72 |

**Table S6.** Different metabolites between Tgy-Q and MC groups in the pathway of biosynthesis of amino acids.

| Metabolites | Tgy-Q vs MC | | | Tgy-Q vs Tgy-C |
|---|---|---|---|---|
| | VIP | Fold Change | P-value | Fold Change |
| L-Arginine | 5.5 | 0.39 | 3.1E-02 | 1.05 |
| L-Histidine | 1.8 | 0.50 | 4.8E-02 | 0.93 |
| L-Tryptophan | 1.7 | 0.62 | 9.0E-02 | 1.08 |
| L-Valine | 1.4 | 0.30 | 1.3E-03 | 0.53 |
| L-Lysine | 2.4 | 0.36 | 2.1E-02 | 1.12 |
| 3-Phosphoserine | 1.3 | 6.40 | 2.5E-02 | 1.43 |
| L-Citrulline | 1.7 | 0.52 | 5.8E-02 | 1.13 |
| L-Threonine | 1.4 | 0.62 | 5.9E-02 | 1.34 |
| L-Glutamine | 1.6 | 0.44 | 6.9E-02 | 0.71 |

**Table S7.** Different metabolites between Tgy-Q and MC groups in the pathway of ABC transporters.

| Metabolites | Tgy-Q vs MC | | | Tgy-Q vs Tgy-C |
|---|---|---|---|---|
| | VIP | Fold Change | P-value | Fold Change |
| N-Acetyl-D-glucosamine | 1.7 | 2.28 | 4.7E-03 | 1.51 |
| Thiamine | 13 | 0.62 | 1.8E-02 | 1.09 |
| L-Arginine | 5.5 | 0.39 | 3.1E-02 | 1.05 |
| L-Histidine | 1.8 | 0.50 | 4.8E-02 | 0.93 |
| Taurine | 1.1 | 0.31 | 7.2E-02 | 1.05 |
| L-Valine | 1.4 | 0.30 | 1.3E-03 | 0.53 |
| L-Lysine | 2.4 | 0.36 | 2.1E-02 | 1.12 |
| L-Threonine | 1.4 | 0.62 | 5.9E-02 | 1.34 |
| D-galacturonic acid | 1.3 | 1.96 | 6.9E-02 | 2.00 |
| L-Glutamine | 1.6 | 0.44 | 6.9E-02 | 0.71 |

**Figure S1.** Tgy-C treatment showed different effect on the metabolome with Tgy-N and Tgy-Q in AD mice. Score scatter plot of OPLS-DA for Tgy-C vs Tgy-N and Tgy-C vs Tgy-Q in positive (A and C) and negative (B and D) ion mode.

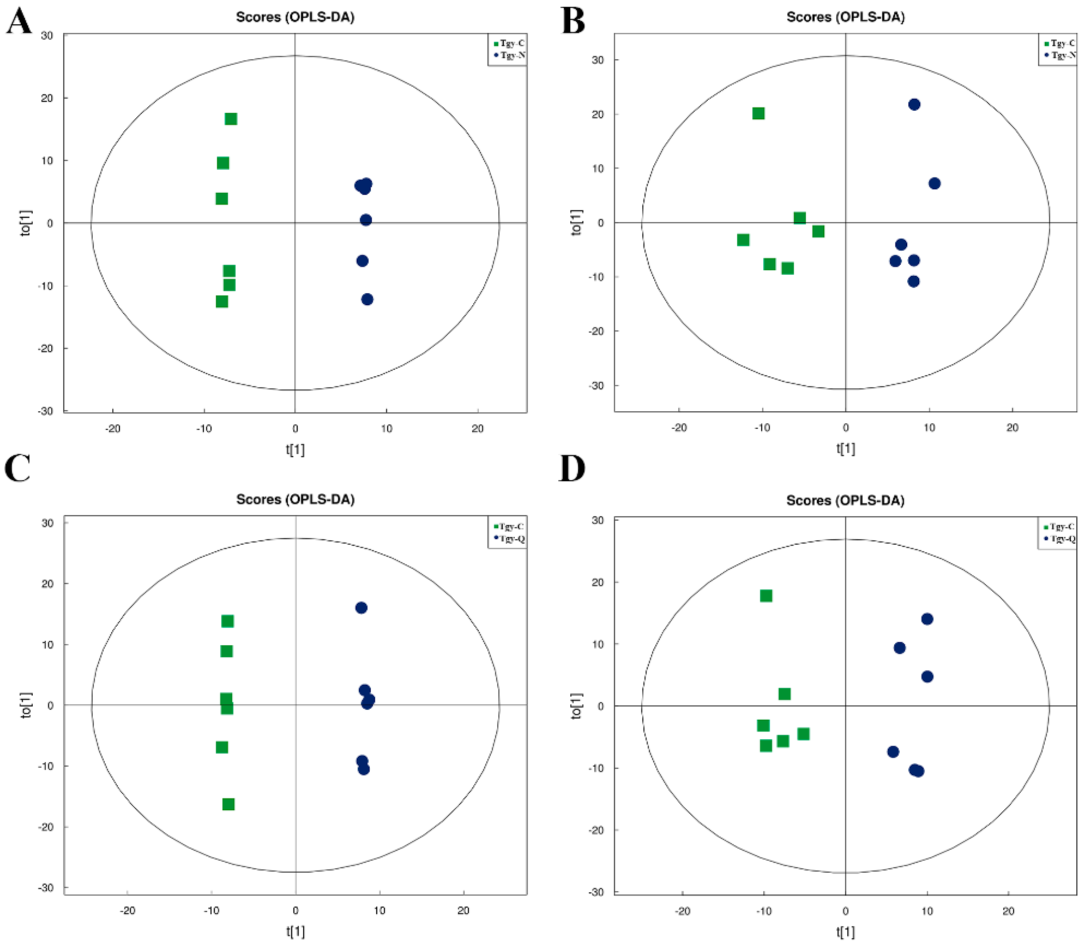

Supplement: Supplementary file 1 [file metabolites-12-00466-s001.zip › metabolites-1719923-supplementary.pdf]
